# Specific nucleotide substitutions in the burst sequence enhance *polyhedrin* expression in alphabaculoviruses: improvement of baculovirus expression vectors

**DOI:** 10.1128/aem.00144-25

**Published:** 2025-04-07

**Authors:** Susumu Katsuma, Koshi Fukaura, Noriko Matsuda-Imai

**Affiliations:** 1 Department of Agricultural and Environmental Biology, Graduate School of Agricultural and Life Sciences, The University of Tokyo 515734 , Tokyo, Japan; University of Nebraska-Lincoln, Lincoln, Nebraska, USA

**Keywords:** polyhedrin, baculovirus, BEVS, burst sequence, BmNPV, AcMNPV

## Abstract

Alphabaculoviruses produce a large number of occlusion bodies (OBs) in host cells during the late stage of infection. OBs are mainly composed of the viral product polyhedrin (POLH), and the extremely high-level transcription of the *polh* gene has been exploited to express foreign proteins in insect cultured cells, larvae, and pupae. This *polh* hyper-transcription requires the “burst sequence” located between the transcriptional start site and the initiation codon. Here, we focused on the roles of the A-rich region within the burst sequence. We generated Bombyx mori nucleopolyhedrovirus mutants whose burst sequence contained “A-to-T” mutations in the A-rich region. Some mutants exhibited levels of *polh* promoter-driven reporter expression lower than or comparable to that of the wild type, whereas the mutants with TTT mutations at positions −16 to −14 in the *polh* upstream region showed a four- to fivefold increase in it. Most cases of single or double A-to-T mutations at –16 to –14 of the upstream region had small but significant effects on the expression level, while the triple mutation was the most effective. This enhancement was also observed in the Autographa californica multiple nucleopolyhedrovirus-based vector system, which is more commonly used for foreign protein expression. We also found that this triple mutation enhanced the accumulation of *polh* mRNA and POLH protein even in an OB-producing virus. These results indicate that specific mutations in the burst sequence have the potential to increase baculoviral protein expression at the transcriptional level and may improve foreign protein expression by baculoviruses.

**IMPORTANCE:**

The most notable characteristic of alphabaculoviruses is that they produce many proteinaceous occlusion bodies (OBs) during the very late stages of infection. The main component of these OBs is virus-encoded polyhedrin (POLH). The high expression of the *polh* gene led to the development of the baculovirus expression vector system (BEVS). Currently, this system is widely used for the production of vaccines, veterinary medicines, and reagents. Despite this background, the mechanisms by which baculoviruses ultimately produce large quantities of OBs remain largely unexplained, even after approximately 40 years since the BEVS development. Here, we discovered that three nucleotide substitutions in the *polh* burst sequence markedly increased the *polh* expression levels in both BmNPV- and AcMNPV-based BEVSs, regardless of the vector type. This discovery can be easily introduced into the currently used BEVS, possibly contributing to further improvements for achieving even higher expression of foreign proteins.

## INTRODUCTION

Baculoviruses are entomopathogenic DNA viruses, which are divided into four genera: *Alphabaculovirus* (lepidopteran-specific nucleopolyhedroviruses [NPVs]), *Betabaculovirus* (lepidopteran-specific granuloviruses), *Gammabaculovirus* (hymenopteran-specific NPVs), and *Deltabaculovirus* (dipteran-specific NPVs) (1). Alphabaculoviruses produce two types of virions: occlusion-derived virus (ODV) and budded virus (BV) during their infection cycle (1). ODVs are embedded in occlusion bodies (OBs), the main component of which is a viral protein called polyhedrin (POLH). OBs are thought to protect ODVs from environmental stresses to which they are exposed under natural conditions, such as UV light and high temperatures. Nucleocapsids are common in ODVs and BVs, but the envelope components are specific to each virion type for establishing the virus infection cycle: ODVs are essential for larva-to-larva viral transmission via oral infection, whereas BVs are used for cell-to-cell infection within an insect host (2, 3).

Alphabaculoviruses produce a huge amount of OBs in the nuclei of infected cells during the late stage of infection (1), which is accomplished by hyperexpression of the *polyhedrin* gene (*polh*). Deletion experiments have revealed that *polh* is not essential for virus replication and can be replaced by a heterologous gene under the strong *polh* promoter in cultured cells (4). The baculovirus expression vector system (BEVS) was developed in the 1980s using Autographa californica multiple nucleopolyhedrovirus (AcMNPV) (5, 6) and Bombyx mori nucleopolyhedovirus (BmNPV) (7) for the expression of foreign proteins in cultured cells and larvae (5–10).

**Fig 1:**
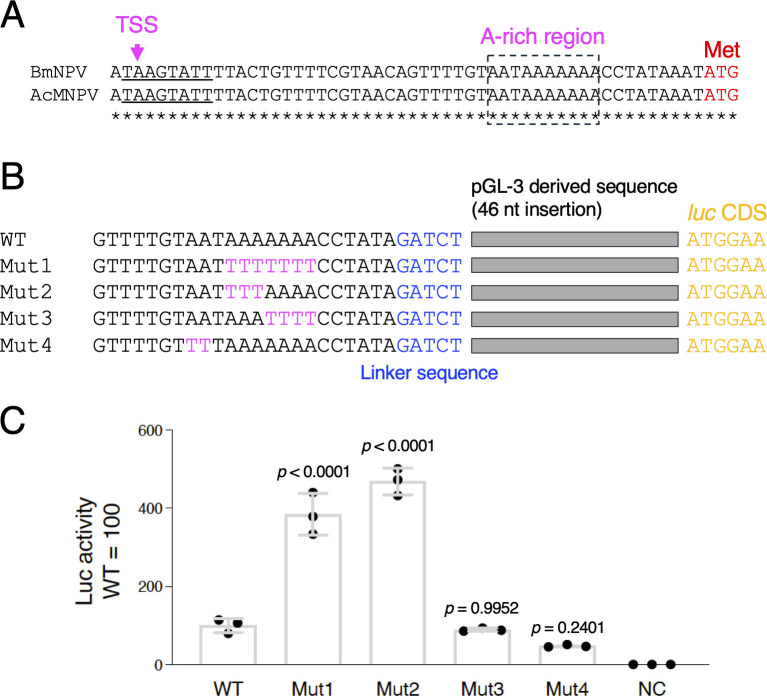
Effects of mutations in the A-rich region of the BmNPV *polh* burst sequence on *polh* promoter-driven activity. (A) Sequence alignment of the burst sequences of BmNPV and AcMNPV. Transcriptional start site (TSS) and A-rich region are indicated by arrow and square with dotted line, respectively. (B) Schematic representation and sequence alignment of the *polh* upstream region of WT, Mut1, Mut2, Mut3, and Mut4. The substituted nucleotides are shown in magenta. (C) Luciferase reporter assay. BmN-4 cells were infected with WT, Mut1, Mut2, Mut3, Mut4, and BmNPV-abb (negative control, NC), and luciferase activity was measured at 3 dpi. Adjusted *P*-values following one-way ANOVA with Tukey’s multiple comparison tests are shown. Data shown are from three independent experiments.

A 50 bplong region located between the transcriptional start site (TSS) and the translational initiation codon of *polh* is called the “burst sequence,” which is essential for the burst of *polh* transcription. Because mutations or deletions in the burst sequence significantly decrease the expression of *polh* (11), the inclusion of the full length of the burst sequence in the *polh*-based transfer/donor vectors used in BEVS has been considered essential. Although the baculovirus protein very late factor 1 (VLF-1) is known to bind to the burst sequence and activate *polh* transcription (11), the nucleotides that are essential for the burst of *polh* expression have not been comprehensively identified. To bridge this knowledge gap, we here focused on the role of the A-rich region within the burst sequence (Fig. 1A). Unexpectedly, we found that specific nucleotide substitutions in the A-rich region markedly enhanced *polh* expression in both BmNPV and AcMNPV. Because the A-rich region is present in most BEVS vectors, this finding may help to improve the currently used BEVS vectors to achieve even higher expression of foreign proteins.

## MATERIALS AND METHODS

### Cell lines and viruses

BmN-4 and Sf-9 cells were cultured at 27°C in TC-100 medium supplemented with 10% fetal bovine serum. The BmN-4 cell line was provided from Susumu Maeda and has been maintained in our laboratory for more than 30 years. The Sf-9 cell line was provided from Ryoichi Sato. BmNPV-abb (12) and T3-WT (13) were used as the parent viruses. Luc-SP (Luc-Bm) (14) and Luc-30 (15), recombinant BmNPVs expressing firefly luciferase driven by the *polh* promoter, were used in the reporter experiments. BmNPV titers were determined by plaque assay on BmN-4 cells (16), and AcMNPV titers were determined by TCID_50_ on Sf-9 cells (17). The cells were infected with BmNPV or AcMNPV at a multiplicity of infection (MOI) of 5.

### Generation of recombinant viruses

To generate recombinant BmNPVs, the *polh* or *luciferase* (*luc*) sequence was cloned into the transfer vector pBm31 (18) using DNA ligase or the In-Fusion HD Cloning Kit (TaKaRa). Mutagenesis was performed using the KOD-Plus mutagenesis kit (Toyobo). DNA sequences were verified by Sanger sequencing. The pBm31 derivatives were cotransfected with Bsu36I-digested BmNPV-abb genomic DNA into BmN-4 cells using X-tremeGENE HP (Roche Applied Science). Recombinants were isolated by plaque assay, and the PCR-amplified *polh* regions were verified by Sanger sequencing.

Recombinant AcMNPVs were generated using the Bac-to-Bac system (Invitrogen) (19). The *luc* or *green fluorescent protein* (*gfp*) sequence was cloned into the transfer vector pFastBac1 using the In-Fusion HD Cloning Kit. Mutagenesis was performed using the KOD-Plus mutagenesis kit. DNA sequences were verified by Sanger sequencing. The generation of bacmid DNAs and recombinant AcMNPVs was performed in accordance with the manufacturer’s protocol.

### Luciferase assay

A luciferase (luc) reporter assay was performed using a luciferase assay system (Promega), as described previously (15). BmN-4 and Sf-9 cells were cultured in a six-well plate and infected with recombinant viruses at an MOI of 5. Luc activity was measured at 3 days post-infection (dpi).

### POLH and GFP accumulation and OB production

BmN-4 or Sf-9 cells were infected with recombinant viruses at an MOI of 5 and harvested at 3 dpi. POLH and GFP expression were examined by sodium dodecyl sulfate-polyacrylamide gel electrophoresis (SDS-PAGE) and gel staining with Coomassie Brilliant Blue (CBB) (13). Stained proteins were detected using a ChemiDoc XRS Plus imaging system (Bio-Rad) and quantified using Image Lab software (Bio-Rad). OB production in BmN-4 cells infected with recombinant viruses at 3 dpi was investigated using a hemocytometer.

### Reverse transcription-quantitative PCR (RT-qPCR)

The BmN-4 and Sf-9 cells were infected with BmNPV and AcMNPV, respectively, at an MOI of 5. The cells were collected at 2 dpi, and total RNA was isolated using TRI Reagent (Sigma-Aldrich). The first-strand cDNA was synthesized from 500 ng of total RNA, and RT-qPCR experiments of *polh*, *luc*, and *gfp* were carried out using the KAPA SYBR FAST qPCR kit (Kapa Biosystems) with the following primers:

T3COpolh-qF: 5′-T(G/T)GGCATGAACAACGAATAC-3′

T3COpolh-qR: 5′-TGTAGAAGTTCTCCCATATG-3′

luc-qF: 5′-TCACTTACGCTGAGTACTTC-3′

luc-qR: 5′-CTGTTGAGCAATTCACGTTC-3′

gfp-qF: 5′-CATCAAGGTGAACTTCAAGATCCG-3′

gfp-qR: 5′-CTCCAGCAGGACCATGTGATC-3′

Amplification was detected using the StepOne real-time PCR system (Applied Biosystems). The expression values were calculated using the 2^–Ct^ method (13).

### Statistics

Statistical analyses were performed using Prism 9 software (GraphPad, USA).

## RESULTS

### Effects of mutations in the A-rich region of the BmNPV *polh* burst sequence on *polh* promoter-driven activity

The burst sequences are completely identical between BmNPV and AcMNPV (Fig. 1A), which are the two major baculoviruses used in BEVS. To investigate in detail the role of the A-rich region within the burst sequence (Fig. 1A, −19 to −10 of the *polh* upstream region), we generated four mutants, Mut1 (−16A to −10A→T), Mut2 (−16A to −14A→T), Mut3 (−13A to −10A→T), and Mut4 (−19A and −18A→T), using a *luc*-expressing BmNPV (Luc-SP, referred to as WT in this paper) (15) as the parent virus. The WT virus contained a 46 bp-long pGL3 vector-derived sequence and a pBm31-derived linker sequence (Fig. 1B). Luc reporter assays using BmN-4 cells revealed that Mut1 and Mut2 exhibited four- to fivefold higher Luc activity, whereas Mut4 and Mut3 showed half and comparable Luc activity compared with the WT virus, respectively (Fig. 1C). These results clearly showed that the substitution of three A nucleotides at −16 to −14 with T enhances the *polh* promoter-driven reporter activity.

### Effects of A-to-T mutation at one or two nucleotides in the A-rich region on *polh* promoter-driven activity

**Fig 2:**
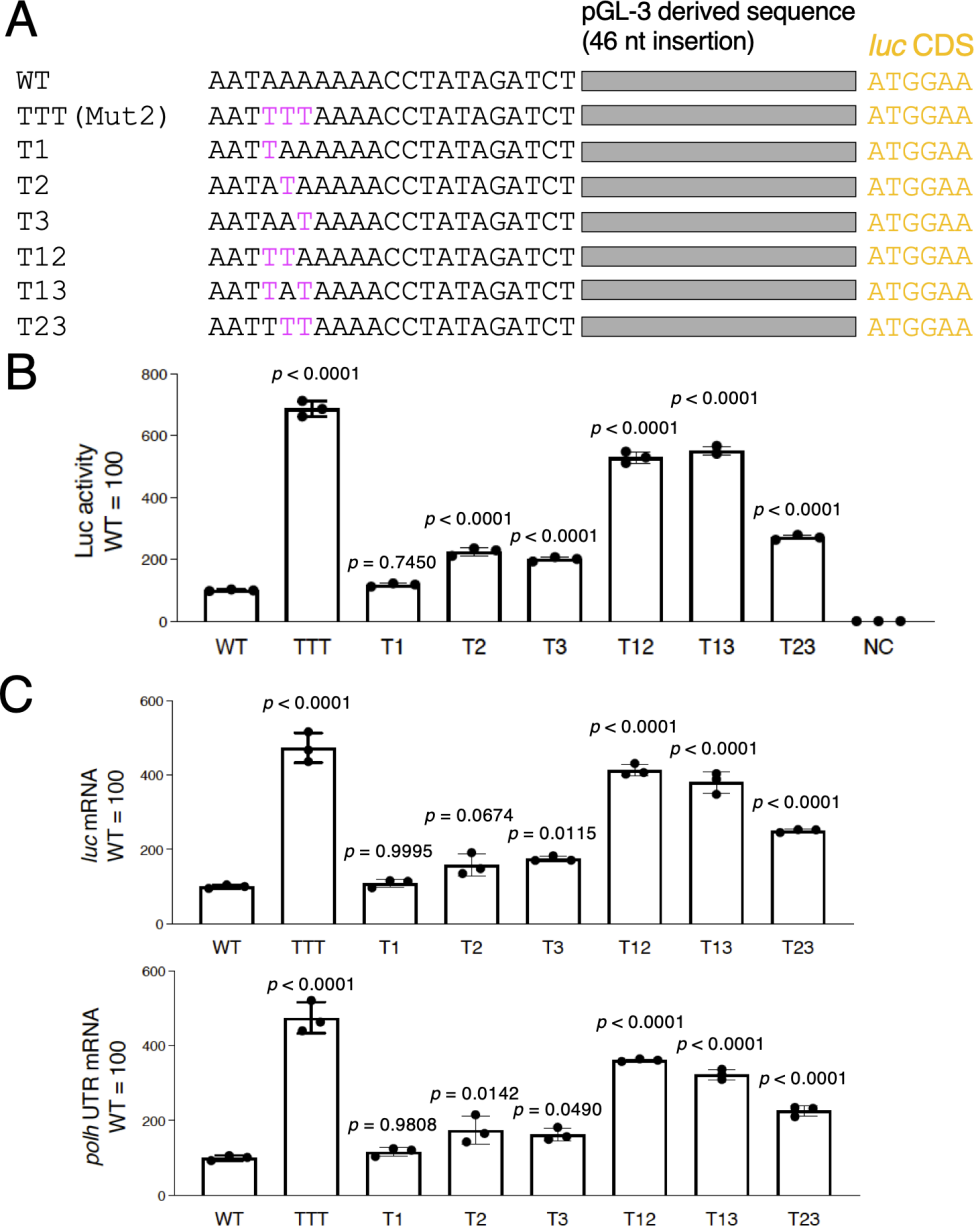
Effects of A-to-T mutation at one or two nucleotides in the A-rich region on *polh* promoter-driven activity. (A) Schematic representation and sequence alignment of the *polh* upstream region of the WT virus and A-to-T mutants. Mut2 in Fig. 1 is hereinafter referred to as TTT. (B) Luciferase reporter assay. BmN-4 cells were infected with WT, A-to-T mutants, and BmNPV-abb (negative control, NC), and luciferase activity was measured at 3 dpi. Adjusted *P*-values following one-way ANOVA with Tukey’s multiple comparison tests are shown. Data shown are from three independent experiments. (C) *luc* expression. Total RNA prepared from BmNPV-infected BmN-4 cells at 2 dpi was subjected to RT-qPCR using the *luc* primer (upper) and the *polh* 3′-UTR primer (lower). Adjusted *P*-values following one-way ANOVA with Tukey’s multiple comparison tests are shown. Data are shown as the mean ± SD from triplicate experiments.

We next investigated which nucleotide exchange among the three nucleotides (−16 to −14) is important for enhanced reporter activity. We generated three mutants with a single A-to-T mutation (T1: −16A to T; T2: −15A to T; T3: −14A to T) and three mutants with two A-to-T mutations (T12: −16A and −15A to T; T13: −16A and −14A to T; T23: −15A and −14A to T) (Fig. 2A). Using these viruses, we measured Luc activity in BmN-4 cells. When a single A was substituted, T1 showed almost no change (*P* = 0.7450) compared with the WT virus, while T2 and T3 showed activity approximately double that of the WT virus (*P* < 0.0001). When two nucleotides were substituted, T12 and T13 showed approximately fivefold activity of the WT virus, and T23 showed approximately double that of the WT virus. However, compared with that of TTT (Mut2), which had all three nucleotides substituted with T, the increase in Luc activity by T12 and T13 was slightly smaller (*P* < 0.0001) (Fig. 2B). We also examined the mRNA levels of *luc* in BmN-4 cells and found a similar trend to the Luc assay results with primer sets targeting *luc* and *polh* 3′-UTR (Fig. 2C). T1 showed almost no change (*P* = 0.9995 in *luc* and *P* = 0.9808 in *polh* 3′-UTR) compared with the WT virus. T2 (*P* = 0.0674 in *luc* and *P* = 0.0142 in *polh* 3′-UTR) and T3 (*P* = 0.0115 in *luc* and *P* = 0.0490 in *polh* 3′-UTR) showed a slight increase. T12 and T13 showed approximately three to four times mRNA level of the WT virus, and T23 showed mRNA level approximately double that of the WT virus. The increase in mRNA in these mutants was lower than that in TTT (T12, *P* = 0.0582 in *luc* and *P* = 0.0002 in *polh* 3′-UTR; T13, *P* = 0.0016 in *luc* and *P* < 0.0001 in *polh* 3′-UTR; T23, *P* < 0.0001 in *luc* and *P* < 0.0001 in *polh* 3′-UTR). Taken together, these results showed that a single-nucleotide substitution resulted in a slight increase in *polh* promoter-driven transcription, two substitutions resulted in a significant increase, and three substitutions maximized the increase.

### Effects of the TTT mutation in the *polh* coding sequence (CDS)-containing vector on *polh* promoter-driven activity

**Fig 3:**
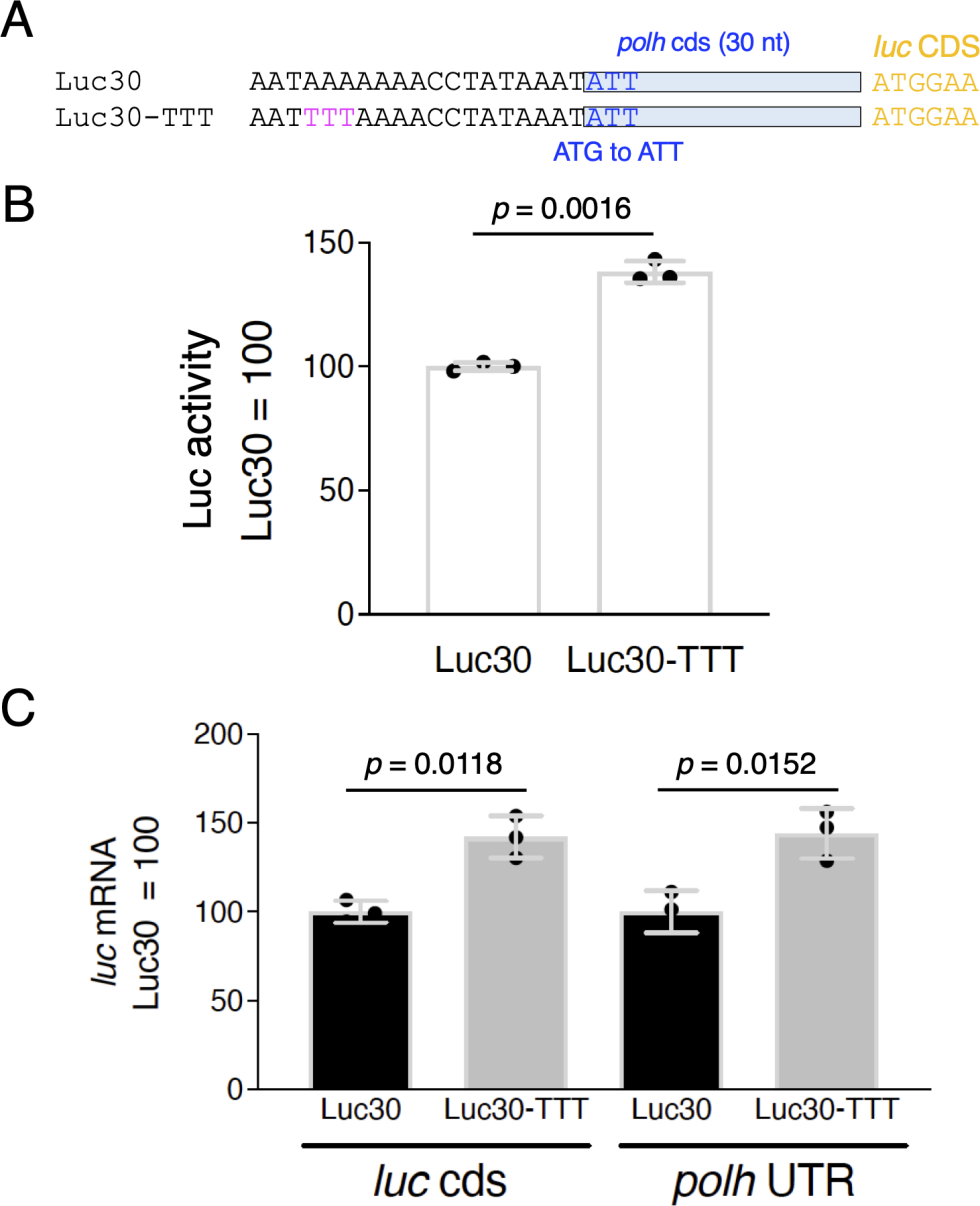
Effects of the TTT mutation in the *polh* CDS-containing vector on *polh* promoter-driven activity. (A) Schematic representation and sequence alignment of the *polh* upstream region of Luc-30 and Luc-30-TTT. Luc-30 contains a 30 bp-long *polh* CDS with a G-to-T substitution at the initiation codon. (B) Luciferase reporter assay. BmN-4 cells were infected with Luc-30 and Luc-30-TTT, and luciferase activity was measured at 3 dpi. Data are shown as the mean ± SD from triplicate experiments. *P*-values were estimated using unpaired *t*-tests with Welch correction (two-tailed). Similar results were obtained in two independent experiments. (C) *luc* expression. Total RNA prepared from BmNPV-infected BmN-4 cells at 2 dpi was subjected to RT-qPCR using the *luc* and the *polh* 3′-UTR primers. Data are shown as the mean ± SD from triplicate experiments. *P*-values were estimated using unpaired *t*-tests with Welch correction (two-tailed). Similar results were obtained in two independent experiments.

Transfer vectors or donor vectors based on the *polh* promoter used in BEVS can be divided into two types: those that include the *polh* CDS (30–37 bp) and those that do not. Luckow and Summers reported that AcMNPV-derived vectors containing 34 or 35 bases of the *polh* CDS achieved higher expression (20). Based on this finding, donor vectors (e.g., pFastBac1) for the Bac-to-Bac system were developed and are now widely used. Recently, we found that the expression of the *polh* promoter-dependent reporter by BmNPV also increased when the 30 bp-long *polh* CDS was included (13). In addition, we reported that the length of the spacer sequence between the burst sequence and the start codon is critical for *polh*-driven reporter expression (15). Since the results of Fig. 1 and 2 were based on vectors with a long spacer and without the CDS (Fig. 1A and B), we investigated whether the TTT mutation would contribute to increased expression even when using a high-level expression vector containing the *polh* CDS with a seamless connection to the reporter gene. We used Luc-30, which includes 30 bp of the BmNPV *polh* CDS (13), as a control and generated Luc-30-TTT by introducing the TTT mutation into the genome of Luc-30 (Fig. 3A). We measured Luc activity using BmN-4 cells infected with Luc-30 or Luc-30-TTT and found that the activity in Luc-30-TTT increased by approximately 1.4 times compared with that of Luc-30 (Fig. 3B). We also examined the *luc* mRNA level by RT-qPCR and found that this level increased by approximately 1.4-fold when the TTT mutation was introduced (Fig. 3C). These results indicate that the TTT mutation increases reporter expression at the mRNA level even in a vector with the 30 bplong *polh* CDS.

### Effects of the TTT mutation in the AcMNPV genome on *polh* promoter-driven activity

**Fig 4:**
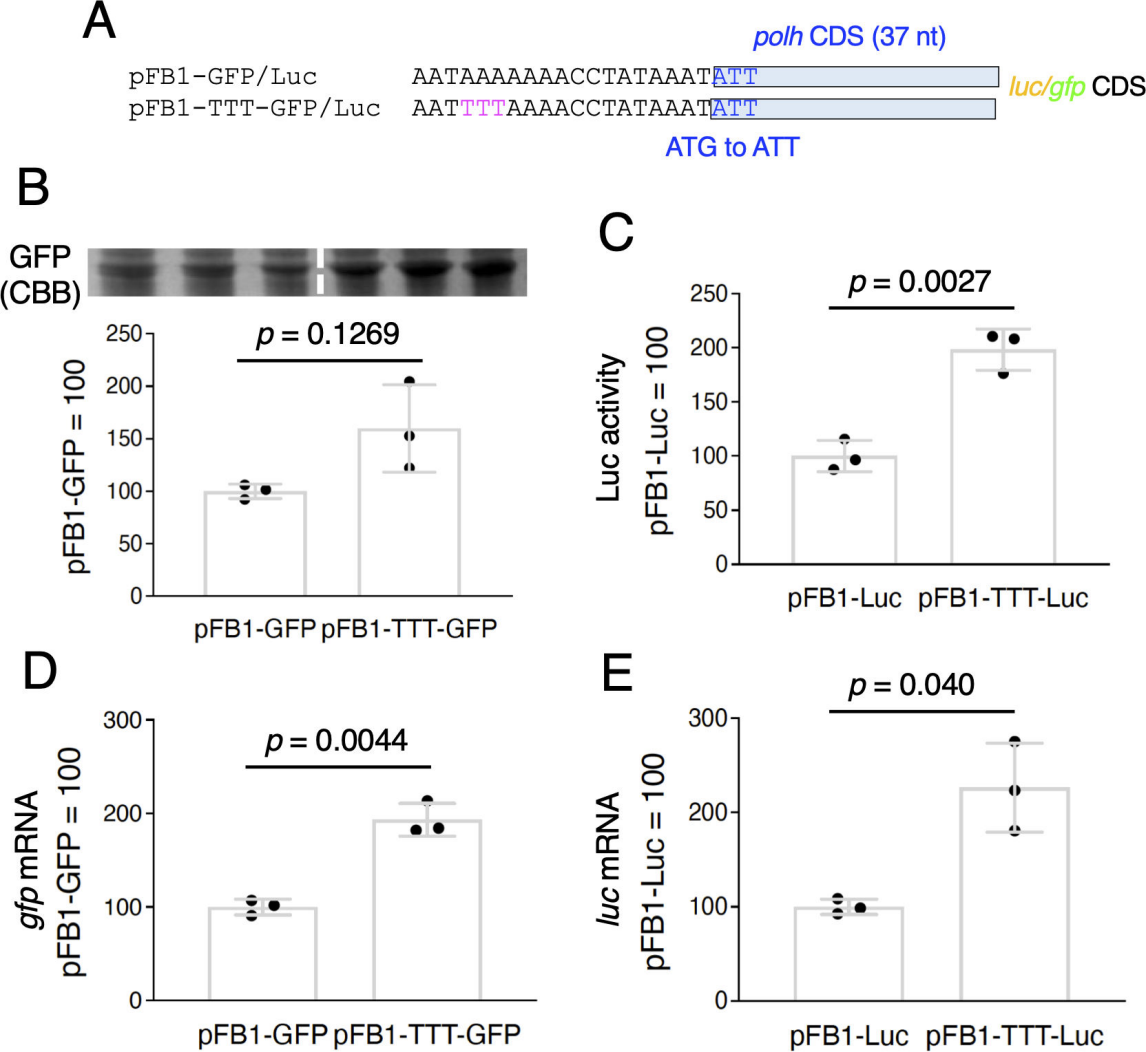
Effects of TTT mutation in the AcMNPV genome on *polh* promoter-driven activity. (A) Schematic representation and sequence alignment of the *polh* upstream region of pFB1-GFP/Luc and pFB1-TTT-GFP/Luc. pFB1 contains a 37 bp-long *polh* CDS with a G-to-T substitution at the initiation codon. (B) GFP expression. Whole cell lysates of AcMNPV-infected Sf-9 cells at 3 dpi were subjected to SDS-PAGE, and the gels stained with CBB are shown. The relative band intensities are shown as the mean ± SD from triplicate experiments. *P*-values were estimated using unpaired *t*-tests with Welch correction (two-tailed). Similar results were obtained in two independent experiments. (C) Luciferase reporter assay. Sf-9 cells were infected with pFB1-Luc and pFB1-TTT-Luc, and luciferase activity was measured at 3 dpi. Data shown are shown as the mean ± SD from triplicate experiments. *P*-values were estimated using unpaired *t*-tests with Welch correction (two-tailed). Similar results were obtained in two independent experiments. *gfp* (**D**) and *luc* (**E**) expression. Total RNA prepared from AcMNPV-infected Sf-9 cells at 2 dpi was subjected to RT-qPCR. Data are shown as the mean ± SD from triplicate experiments. *P*-values were estimated using unpaired *t*-tests with Welch correction (two-tailed). Similar results were obtained in two independent experiments.

AcMNPV-based BEVS has been more commonly used than the BmNPV-based system. We thus next investigated the impact of the TTT mutation on AcMNPV using the Bac-to-Bac system. We used pFastBac1 as the donor vector, which included the 37 bplong *polh* CDS, and *luc* and *gfp* as reporter genes (Fig. 4A). We first examined GFP expression levels in Sf-9 cells infected with each recombinant AcMNPV (pFB1-GFP and pFB1-TTT-GFP). As shown in Fig. 4B, GFP expression increased by approximately 1.5 times (not statistically significant, *P* = 0.1269) with the introduction of the TTT mutation. We also measured the Luc activity upon infection with pFB1-Luc and pFB1-TTT-Luc. The Luc assay results showed that introducing the mutation increased the activity by approximately two times compared with that in the control (Fig. 4C). Moreover, we observed a significant increase in *gfp* and *luc* mRNA by the introduction of the TTT mutation (Fig. 4D and E). These results showed that the TTT mutation also enhances the *polh*-driven expression of foreign genes in the AcMNPV-Sf-9 system.

### Effects of TTT mutation on *polh* expression in an OB-producing BmNPV

**Fig 5:**
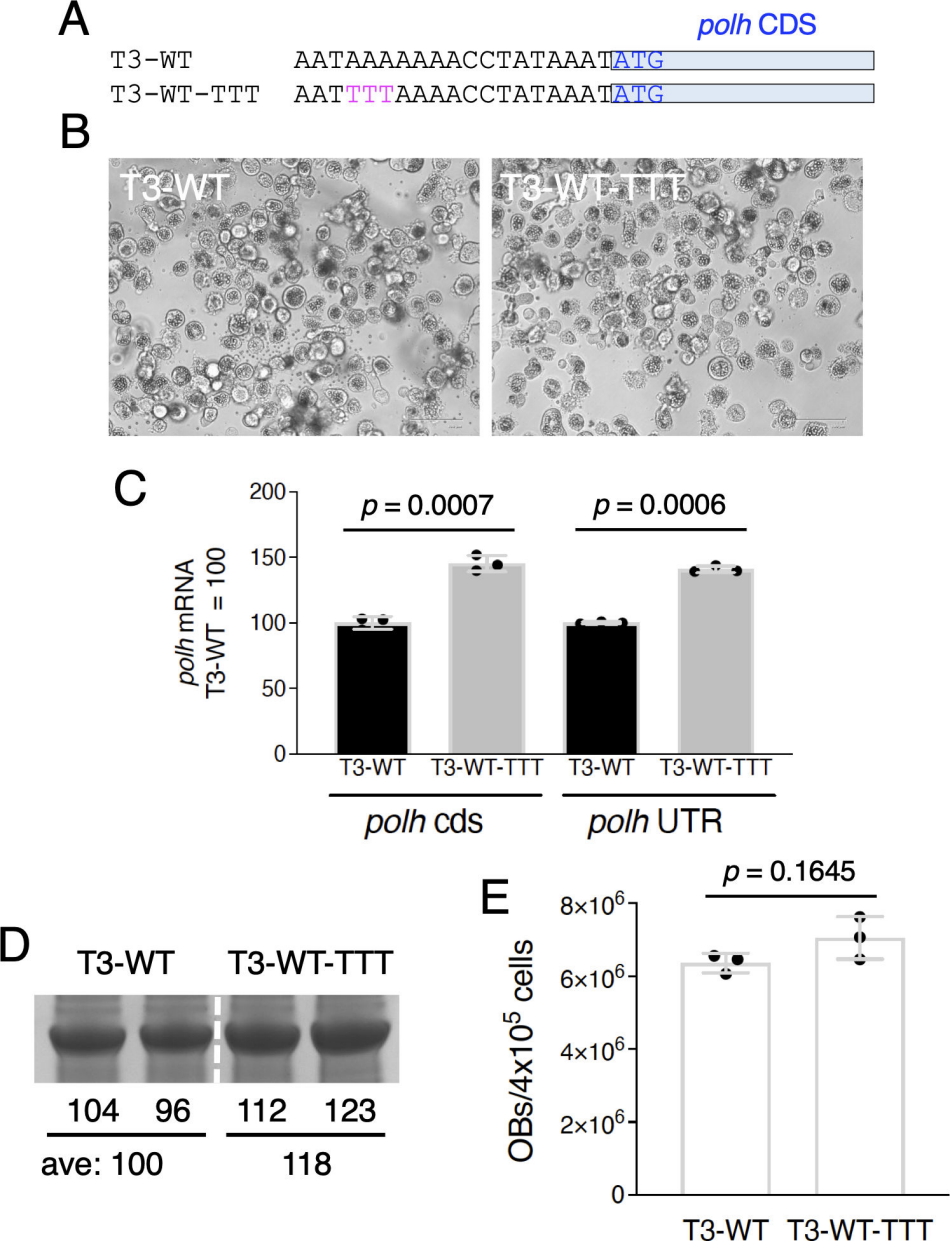
Effects of TTT mutation on *polh* expression in an OB-producing BmNPV. (A) Sequence alignment of the *polh* upstream region of T3-WT and T3-WT-TTT. (B) Light microscopy observations of T3-WT- and T3-WT-TTT-infected BmN-4 cells at 3 dpi. Scale bar = 100 µm. (C) *polh* expression. Total RNA prepared from BmNPV-infected BmN-4 cells at 2 dpi was subjected to RT-qPCR using *polh* CDS and 3′-UTR primers. Data are shown as the mean ± SD from triplicate experiments. *P*-values were estimated using unpaired *t*-tests with Welch correction (two-tailed). Similar results were obtained in two independent experiments. (D) POLH accumulation. Whole cell lysates of BmNPV-infected BmN-4 cells at 3 dpi were subjected to SDS-PAGE, and the gels stained with CBB are shown. The relative band intensities are also shown. (E) Number of OBs produced in BmN-4 cells infected with T3-WT or T3-WT-TTT at 3 dpi. *P*-values were estimated using unpaired *t*-tests with Welch correction (two-tailed). Similar results were obtained in two independent experiments.

We next investigated whether *polh* expression would be affected by introducing the TTT mutation into the genomes of an OB-producing virus. We used an OB-producing BmNPV T3-WT as the parent virus (13) and a virus with the TTT mutation (T3-WT-TTT, Fig. 5A). T3-WT possesses the same sequences of the wild-type BmNPV *polh*. Both viruses formed large numbers of OBs within the nuclei of infected cells at the very late stage of infection (Fig. 5B). RT-qPCR results showed that the introduction of the TTT mutation increased the *polh* mRNA level by approximately 1.4 times (Fig. 5C). We also performed SDS-PAGE to examine POLH expression levels and found that this expression increased by about 1.2 times with the introduction of the TTT mutation (Fig. 5D). T3-WT-TTT produced more OBs than T3-WT, but no statistically significant difference was observed (Fig. 5E). These results indicate that the introduction of the TTT mutation can induce an increase in *polh* expression even in the virus with the wild-type *polh* gene.

## DISCUSSION

In our previous research, we identified the length of the spacer between the burst sequence and the start codon (15) and the 30 bp-long 5′ *polh* CDS (13) as the critical factors for maximizing the expression of foreign genes using BEVS. In this study, we focused on the A-rich region within the *polh* burst sequence because a study on other viruses suggested that A-rich (poly-A) sequences upstream of viral genes contribute to their translation (21). The *polh* burst sequences of AcMNPV and BmNPV contain an identical A-rich sequence, and linker scanning mutagenesis at positions −23 to −14 of the burst sequence (with substitution of six out of nine nucleotides of the A-rich region) completely diminished the *polh*-driven reporter activity in AcMNPV (11), indicating the importance of this region. In this study, we showed that the *polh*-driven reporter expression decreased in Mut4, supporting the importance of the burst sequence in *polh* expression. Conversely, mutations of nucleotides AAA to TTT at positions −16 to −14 of the burst sequence were found to increase reporter expression by four- to fivefold. This increase was observed in both BmNPV and AcMNPV, regardless of the vector type, and surprisingly, it was also observed in an OB-producing BmNPV. Given these findings, there is a need to investigate the advantages and disadvantages of this triple mutation in wild-type viruses in the wild, although we did not observe any disadvantages of them under *in vitro* conditions (cultured cells).

In this study, we have not yet elucidated the cause of the increase in *polh* mRNA levels achieved by introducing the TTT mutation. It is known that VLF-1 binds to the burst sequence, which is considered essential for the high-level expression of *polh* (11). Therefore, it is possible that the TTT mutation increases the binding affinity of VLF-1 to the burst sequence, thereby increasing the *polh* transcription level. In future work, it will be necessary to examine this possibility using methods evaluating DNA–protein binding, such as gel shift assay or modified chromatin immunoprecipitation assay.

Currently, almost all available BEVs employ either BmNPV or AcMNPV, and their transfer vectors and donor vectors contain burst sequences. While a previous study reported the introduction of multiple copies of burst sequences into transfer vectors (22), there have been no reports of an increase in *polh*-driven reporter expression resulting from simple nucleotide substitutions within the burst sequence. The A-rich sequences are present in almost all BEVS, and the TTT mutation can be easily introduced into the currently used BEVS. Although we have not examined whether the TTT mutation also enhances recombinant protein production in larval insects, this discovery may contribute to further improvements in productivity using BEVS, which is applied industrially for producing vaccines and veterinary medicines.

## References

[1] Rohrmann GF. 2019. Baculovirus Molecular Biology. 4th ed. National Library of Medicine, Bethesda, MD.

[2] Granados RR, Lawler KA. 1981. In vivo pathway of Autographa californica baculovirus invasion and infection. Virology (Auckl) 108:297–308. doi:10.1016/0042-6822(81)90438-4 18635031

[3] Keddie BA, Aponte GW, Volkman LE. 1989. The pathway of infection of Autographa californica nuclear polyhedrosis virus in an insect host. Science 243:1728–1730. doi:10.1126/science.2648574 2648574

[4] Smith GE, Fraser MJ, Summers MD. 1983. Molecular engineering of the Autographa californica nuclear polyhedrosis virus genome: deletion mutations within the polyhedrin gene. J Virol 46:584–593. doi:10.1128/JVI.46.2.584-593.1983 16789242 PMC255161

[5] Smith GE, Summers MD, Fraser MJ. 1983. Production of human beta interferon in insect cells infected with a baculovirus expression vector. Mol Cell Biol 3:2156–2165. doi:10.1128/mcb.3.12.2156-2165.1983 6318086 PMC370086

[6] Pennock GD, Shoemaker C, Miller LK. 1984. Strong and regulated expression of Escherichia coli beta-galactosidase in insect cells with a baculovirus vector. Mol Cell Biol 4:399–406. doi:10.1128/mcb.4.3.399-406.1984 6325875 PMC368716

[7] Maeda S, Kawai T, Obinata M, Fujiwara H, Horiuchi T, Saeki Y, Sato Y, Furusawa M. 1985. Production of human alpha-interferon in silkworm using a baculovirus vector. Nature 315:592–594. doi:10.1038/315592a0 2989694

[8] Ardisson-Araújo DMP, Rocha JR, da Costa MHO, Bocca AL, Dusi AN, de Oliveira Resende R, Ribeiro BM. 2013. A baculovirus-mediated strategy for full-length plant virus coat protein expression and purification. Virol J 10:262. doi:10.1186/1743-422X-10-262 23945471 PMC3765376

[9] Mc Callum GJ, Arregui MB, Smith I, Bracco LF, Wolman F, Cascone O, Targovnik AM, Miranda MV. 2019. Recombinant protein purification in baculovirus-infected Rachiplusia nu larvae: an approach towards a rational design of downstream processing strategies based on chromatographic behavior of proteins. Protein Expr Purif 158:44–50. doi:10.1016/j.pep.2019.02.009 30772376

[10] Marumoto Y, Sato Y, Fujiwara H, Sakano K, Saeki Y, Agata M, Furusawa M, Maeda S. 1987. Hyperproduction of polyhedrin-IGF II fusion protein in silkworm larvae infected with recombinant Bombyx mori nuclear polyhedrosis virus. J Gen Virol 68 (Pt 10):2599–2606. doi:10.1099/0022-1317-68-10-2599 3312489

[11] Yang S, Miller LK. 1999. Activation of baculovirus very late promoters by interaction with very late factor 1. J Virol 73:3404–3409. doi:10.1128/JVI.73.4.3404-3409.1999 10074194 PMC104104

[12] Zhou CE, Ko R, Maeda S. 1998. Polyhedron-like inclusion body formation by a mutant nucleopolyhedrovirus expressing the granulin gene from a granulovirus. Virology (Auckl) 240:282–294. doi:10.1006/viro.1997.8927 9454702

[13] Katsuma S, Matsuda-Imai N. 2024. Codon optimization-based whole-gene scanning identifies hidden nucleotides essential for Bombyx mori nucleopolyhedrovirus polyhedrin hyperexpression. J Mol Biol 436:168595. doi:10.1016/j.jmb.2024.168595 38724003

[14] Nakanishi T, Goto C, Kobayashi M, Kang W, Suzuki T, Dohmae N, Matsumoto S, Shimada T, Katsuma S. 2010. Comparative studies of lepidopteran baculovirus-specific protein FP25K: development of a novel Bombyx mori nucleopolyhedrovirus-based vector with a modified FP25K gene. J Virol 84:5191–5200. doi:10.1128/JVI.00099-10 20219904 PMC2863835

[15] Katsuma S, Matsuda-Imai N. 2023. A seamless connection from the burst sequence to the start codon is essential for polyhedrin hyperexpression in alphabaculoviruses. Biochem Biophys Res Commun 679:1–5. doi:10.1016/j.bbrc.2023.08.046 37651871

[16] Maeda S. 1984. A plaque assay and cloning of Bombyx mori nuclear polyhedrosis virus. J Seric Sci Jpn 53:547–548.

[17] Reed LJ, Muench H. 1938. A simple method of estimating fifty-percent endpoints. Am J Hyg 27:493–497. doi:10.1093/oxfordjournals.aje.a118408

[18] Ko R, Okano K, Maeda S. 2000. Structural and functional analysis of the Xestia c-nigrum granulovirus matrix metalloproteinase. J Virol 74:11240–11246. doi:10.1128/jvi.74.23.11240-11246.2000 11070022 PMC113222

[19] Katsuma S, Daimon T, Horie S, Kobayashi M, Shimada T. 2006. N-linked glycans of Bombyx mori nucleopolyhedrovirus fibroblast growth factor are crucial for its secretion. Biochem Biophys Res Commun 350:1069–1075. doi:10.1016/j.bbrc.2006.10.001 17046716

[20] Luckow VA, Summers MD. 1989. High level expression of nonfused foreign genes with Autographa californica nuclear polyhedrosis virus expression vectors. Virology (Auckl) 170:31–39. doi:10.1016/0042-6822(89)90348-6 2497580

[21] Jha S, Rollins MG, Fuchs G, Procter DJ, Hall EA, Cozzolino K, Sarnow P, Savas JN, Walsh D. 2017. Trans-kingdom mimicry underlies ribosome customization by a poxvirus kinase. Nature 546:651–655. doi:10.1038/nature22814 28636603 PMC5526112

[22] Kato T, Manohar SL, Kanamasa S, Ogata M, Park EY. 2012. Improvement of the transcriptional strength of baculovirus very late polyhedrin promoter by repeating its untranslated leader sequences and coexpression with the primary transactivator. J Biosci Bioeng 113:694–696. doi:10.1016/j.jbiosc.2012.01.010 22309650

